# Plague in Iran: its history and current status

**DOI:** 10.4178/epih.e2016033

**Published:** 2016-07-24

**Authors:** Abdolrazagh Hashemi Shahraki, Elizabeth Carniel, Ehsan Mostafavi

**Affiliations:** 1 Department of Epidemiology, Pasteur Institute of Iran, Tehran, Iran; 2 National Reference Laboratory for Plague, Tularemia, and Q fever, Research Centre for Emerging and Reemerging Infectious Diseases, Pasteur Institute of Iran, Akanlu, Kabudar-Ahang, Hamadan, Iran; 3 Yersinia Research Unit, National Reference Laboratory, Institut Pasteur, Paris, France

**Keywords:** Middle East, Iran, History of medicine, *Yersinia pestis*

## Abstract

**OBJECTIVES:**

Plague remains a public health concern worldwide, particularly in old foci. Multiple epidemics of this disease have been recorded throughout the history of Iran. Despite the long-standing history of human plague in Iran, it remains difficult to obtain an accurate overview of the history and current status of plague in Iran.

**METHODS:**

In this review, available data and reports on cases and outbreaks of human plague in the past and present in Iran and in neighboring countries were collected, and information was compiled regarding when, where, and how many cases occurred.

**RESULTS:**

This paper considers the history of plague in Persia (the predecessor of today’s Iran) and has a brief review of plague in countries in the World Health Organization Eastern Mediterranean Region, including a range of countries in the Middle East and North Africa.

**CONCLUSIONS:**

Since Iran has experienced outbreaks of plague for several centuries, neighboring countries have reported the disease in recent years, the disease can be silent for decades, and the circulation of *Yersinia pestis* has been reported among rodents and dogs in western Iran, more attention should be paid to disease monitoring in areas with previously reported human cases and in high-risk regions with previous epizootic and enzootic activity.

## INTRODUCTION

Plague is a life-threatening infectious disease that is still endemic in some areas around the world. It is caused by the bacillus *Yersinia pestis*, which was discovered in 1894 by Alexandre Yersin [1]. The natural reservoir of this disease is wild rodents, and the causative agent of the disease is transmitted to different animals and humans via infected fleas [2,3]. The most prominent infection vector for humans is the *Xenopsylla* genus, in particular *X. cheopis*. Living in rural areas, hunting or trapping animals, and keeping cats and dogs increase the risk of infection among humans [2]. The disease in humans exists in three clinical forms: bubonic, pneumonic, and septicemic. Without treatment, the mortality may be as high as practically 100%, particularly for pneumonic plague [4].

As *Y. pestis* circulates at low levels in the rodent population in endemic foci of the disease [5], outbreaks are always possible, and a timely diagnosis is necessary for effective treatment [4,6]. *Y. pestis* has also been used in bioterrorism in the past and its usage in the future for such purposes is not difficult to imagine [7]. Studies have shown not only a trend of decreasing susceptibility to some antibiotics, but also the emergence of multi-drug-resistant strains [8,9], which underscores the need for plague to receive more attention as a significant public health concern than has previously been the case.

There have been three great world pandemics of plague. The first one, known as the Justinian plague, occurred in the Byzantine Empire (Constantinople) in the 6th century Common Era (CE) and spread through the Middle East, the Mediterranean, and Europe. The second pandemic, known as the Black Death, began in India, China, and some regions of Russia, and reached Western Europe in 1347 CE. The third pandemic was the Hong Kong epidemic that started in 1855 CE in Yunnan Province, China [2,4]. *Y. pestis* has been reported throughout the world, and plague is enzootic in rodents in Africa, North and South America, and Asia (the Middle East, Far East, and countries of the former Soviet Union) [2,10,11]. According to the International Health Regulations, the pneumonic form of the disease must be reported to the World Health Organization (WHO) immediately [12].

Some plague endemic foci still exist in central Asia; western Arabia (the Asir region); the Middle East, with a center in Iranian Kurdistan; central and southern Africa, and northwestern India [13-16]. Between 1987 and 2001, 36,876 confirmed cases of plague with 2,847 deaths were reported to the WHO [4, 15]. In 2013, 783 cases were reported worldwide, resulting in 126 deaths [17]. In recent years, more than 95% of human cases have been reported in Africa, mainly in Madagascar [18]. Plague has a seasonal pattern in most endemic areas, which is associated with the predominant vectors and rodent reservoirs and their ecology in specific geographical niches [4]. In the beginning of the 1990s, plague was believed to have been eliminated due to the paucity of reported cases. However, in 1994, India experienced a large outbreak of pneumonic plague after 30 years with no reports of the disease [13], and in 2003, an outbreak of plague was reported in Algeria, in an area considered plague-free for 50 years [14]. The reemergence of plague was reported in Libya in 2009, after 25 years without a case of plague [19]. In August 2013, in a region of Kyrgyzstan bordering Kazakhstan, a case of bubonic plague was reported [20].

Plague is one of the oldest infectious diseases in Iran, and has had devastating effects on the human population of Iran throughout history. Our understanding of plague pandemics has rapidly expanded in the last decade, largely due to molecular approaches, but much remains to be understood regarding past plague epidemics in various parts of the world, particularly in Iran, as Persia was the greatest empire in the Middle East for much of its history.

The region of Kurdistan, north of the Zagros Mountains in Iran and stretching across southern Turkey and the north of Iraq and Syria, is considered one of the most significant endemic foci of plague worldwide [21-25].

This review considers the history of plague in Persia (the predecessor of today’s Iran) and incorporates a brief review of the countries in the WHO Eastern Mediterranean Region, consisting of several countries in the Middle East and North Africa that have frequently experienced plague outbreaks in recent decades, including Afghanistan, Bahrain, Iraq, Jordan, Kuwait, Lebanon, Saudi Arabia, Syria, United Arab Emirates, Yemen, Egypt, Libya, Morocco, and Tunisia.

## GEOGRAPHY OF PERSIA/IRAN

Historically, the territory of the Persian Empire encompassed all of today’s Iran, Iraq, Lebanon, Jordan, Armenia, Turkey, and Syria, as well as parts of Saudi Arabia, Afghanistan, Pakistan, the Caucasus, Central Asia, and Egypt.

Iran is a Middle Eastern country, located south of the Caspian Sea and north of the Persian Gulf and Gulf of Oman. It shares borders with Iraq, Turkey, Azerbaijan, Turkmenistan, Armenia, Afghanistan, and Pakistan. With an area of 1,648,000 km^2^ (636,000 square miles), Iran ranks eighteenth in size among the countries of the world. Iran has a variable arid climate, in which most of the relatively scant annual precipitation falls between October and April. Seven percent of the country is forested.

## PLAGUE IN IRAN

Historical records show that plague has been active in Iran for centuries; however, insufficient information, invalid documentation, the misclassification of plague as a different infectious disease such as cholera, and the way in which plague has interacted with social dynamics have hindered the analysis of plague outbreaks over time.

**Figure 1.**
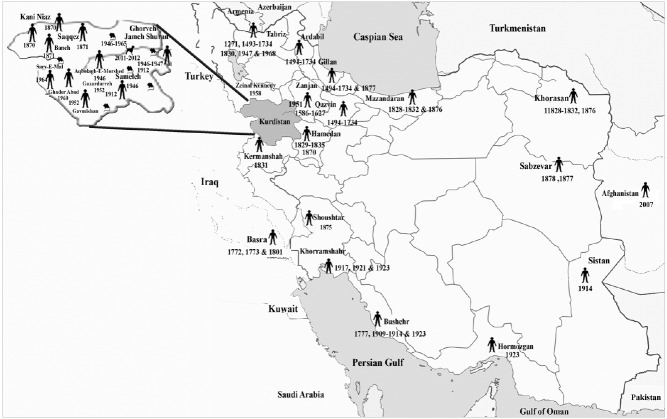
Areas affected by plague throughout the history of Iran. Kurdistan, known to be the focus of plague in Iran, has been drawn larger.

Plague frequently recurred in Iran throughout history, but the dating and characterization of the nature of plague outbreaks remain imprecise. The rapid expansion of plague with high mortality rates may have been related to sparse treatment centers, a poor public health system, the absence of effective quarantine arrangements, washing the dead bodies in rivers before burial, and transferring corpses to sacred places such as Mashhad, Qom, Najaf, and Karbala [26]. Plague epidemics in Iran typically originated from villages or places with poor hygiene, and rural epidemics have been found to last for periods of 30 to 40 years with no officially reported cases [27]. Throughout the course of history, almost all regions of the country, especially near the borders (in the north, south, east, and west) have reported plague outbreaks (Figure 1, Table 1).

In 543 CE, plague reached what is now modern Iran after passing from Italy to Syria, Palestine, and Iraq, and infected the Persian imperial army and the population at that time [28]. In 544 CE, the plague infected the army of the Roman Empire and the army of the Persian Empire at a time when they were at war [29]. In 627 CE, a large epidemic of plague, which led to the death of more than 100,000 people in Ctesiphon, the Sassanian capital, close to Baghdad, was reported. Shortly thereafter, Shiroyeh, the king of Persia, died of plague [30]. Another plague epidemic occurred from 634 to 642 CE in the region of Yezdigird III, the ‘great king’ of Persia [31]. The Yezdigird Plague may be another name for the plague epidemic in Syria and Palestine that is known as the Amwas (or Amawas) Plague, which killed almost 25,000 people in 638 or 639 CE [32]. The Amwas Plague is considered one of the numerous outbreaks of plague in the sixth, seventh, and eighth centuries that followed the major pandemic of the sixth century, the Justinian Plague [33]. In 688-689 CE, a devastating plague swept through Basra and killed 70,000 to 73,000 people [31]. The Persian Empire experienced considerable damage from plague outbreaks to both its population and its military. Its weakened military strength is considered a factor contributing to the inability of Persia to prevent the Arab conquest of its territory [34].

To the best of our knowledge, no concrete documentation exists of plague outbreaks and the impact of the plague on Persia between 689 and 1270 CE; it seems, though, that plague continued to spread throughout Persia, remaining endemic after the outbreaks of 689 until the middle of the thirteenth century. Ali ibn al-Abbas al-Majusi (933-1000 CE) described plague in his medical book titled *Kitāb al-Malakiyy*. Abu Sahl ‘Isa ibn Yahya al-Masihi al-Jorjani (960-1000 CE) wrote an article about plague; and, interestingly, the *Canon of Medicine* of *Avicenna* (980-1037 CE) noted the clinical signs of bubonic plague and Esmail Jorjani (1042-1137 CE) mentioned inguinal lymphadenopathy as a sign of bubonic plague [35].

Marco Polo, the famous Italian merchant traveler, reached the city of Tabriz, in northwestern Iran, in 1270 and in *The Travels of Marco Polo*, he mentioned that the city gates were closed due to the plague [36].

Several outbreaks of human plague occurred during the Safavid period (1495-1735 CE), affecting areas in northern Iran such as Gilan, Tabriz, Qazvin, and Ardabil. Another outbreak occurred in Qom, another city in the north, which continued for five years with 12,000 deaths. After the collapse of the Safavid Dynasty and the occupation of Iran by Afghan invaders, another plague outbreak was recorded in Gilan in 1727 CE. In 1731 CE, approximately 20,000 people died due to a plague outbreak in Hamadan and the western region of Iran [37].

In the epidemics of 1772-1773 CE in Iran, quarantine practices were introduced into the Persian Gulf region for first time [38]. This outbreak is recorded as one of the most severe epidemics of plague, killing an estimated two million people in Persia (Iran) and Persian-controlled lands to the west. Plague was introduced to Baghdad, today the capital of Iraq, in the winter of 1772 and reached Basra in 1773. The plague killed more than 250,000 people in Basra alone. A thousand deaths were recorded daily, and the disease spread to Bombay, India [38]. Plague also spread southward along the Persian Gulf to Bushehr, expanding over most parts of Persia and reaching Bahrain in 1773 [38]. In 1798, a small outbreak of the plague was recorded in Mosul [38].

In 1800, a plague outbreak started in the city of Mosul, northern Iraq, and spread to Baghdad and Istanbul. The authorities introduced quarantine measures to prevent the spread of the plague to India. During this wave, the workers of the British East India Company were moved to a village outside Basra. This led to no reports of plague among European residents [38].

In 1830, an epidemic of plague affected the entire Persian Gulf region. The disease began in the fall of 1830 with an outbreak in Tabriz, northern Iran, which led to the death of 30,000 people and the relocation of the Iranian capital from Tabriz to Ardabil [26,38]. In 1831, the disease reached northern Iran and lead to the death of thousands of people in Mazandaran and Gilan (Rasht) [26]. This plague outbreak reduced the population of Rasht from 60,000 to 15,000-20,000 [26,39].

A plague epidemic in western Iran, in the Kurdistan, Kermanshah, and Hamadan regions, led to thousands of deaths from 1829 to 1835 and in 1870 [27]. In 1876, a plague epidemic in Shushtar, southwestern Iran, led to 1,800 deaths out of a population of 8,000 within only a few months [40]. The plague was again reported in Gilan and had a significant negative impact on the economy [41]. An outbreak of plague in villages including Serin Bulagh, Saghez, and Baneh in Kurdistan, western Iran, killed hundreds of people [42,43]. At the time, Italian doctors came from Istanbul to provide assistance with the plague epidemic in Persia [44]. Moreover, in 1877, plague recurred around Khorasan, eastern Iran, and around the Caspian Sea [42].

Mohammad Razi Tabatabai, the senior physician of the army during the reign of King Naser al-Din Shah Qajar wrote a book on plague in 1875, in which he described the medical practices of the time and discussed control strategies for plague [45]. Between 1870 and 1882, Dr. Theodorides documented the places in Kurdistan where plague commonly occurred, and noticed certain villages to be foci of the disease in this region [46].

A plague epidemic in a village north of Sabzevar, Khorasan Province in northwest Iran killed 37 individuals in 1877 [26]. Another epidemic in 1878, in two neighboring villages in Khorasan, left hundreds dead [27].

In 1877, an epidemic of the disease was again reported in the cities of Rasht, Bushehr, and Kermanshah. Following that, a devastating epidemic occurred in the regions around the Persian Gulf; as a result, British forces founded the first quarantine center on Hengam Island, Bushehr, to prevent further transmission of the disease to other points of the country [47].

The plague epidemic in 1906 in the province of Sistan and Baluchestan, southeastern Iran, was restricted to the regions around Sistan Lake, and it is believed that the infection was transmitted to this area via old clothes imported from India [44].

During the plague epidemics between 1910 and 1911 in Bushehr, in southern Iran, people fled the city, leading to a negative economic impact [44]. In 1912 and 1913, the plague reemerged in Bushehr; 750 people died and 4,000 vaccinations were conducted at that time. Bushehr was then spared from the disease until 1924 when the next outbreak occurred [44]. Most cases of the disease in the Persian Gulf region happened in the first quarter of the 20th century, and there is a high probability that the disease originated from India [41,49].

In 1913, outbreaks were seen in two provinces in Iran: one in Kurdistan in the west, and the other in Khorasan, in the east [22]. A plague outbreak was reported in Khorramshahr and Abadan, in southwestern Iran, in 1924 [44]. The disease reemerged in Torbat-e Jam, in Khorasan, after eight years without a reported case [24].

Some of the plague outbreaks occurred in the area where the Anglo-Persian Oil Company, an English company, was actively extracting petroleum in the southern part of the country. The measures that were taken by this company had a great impact on the control of plague in the area. In 1925, plague vaccination started in the southern areas of Iran and 4,553 individuals were vaccinated. Houses were disinfected and with any new report of the disease, serious actions were taken, such as disinfection of all clothing, isolation of the patient, destruction of the house (with recompense), and prohibition of rebuilding on that spot [38].

## PLAGUE IN IRAN OVER THE LAST CENTURY

Plague was not reported again for 23 years until an outbreak in Kurdistan, western Iran, in 1947 [24]. Due to the presence of the appropriate species of wild rats and fleas in Kurdistan, this region had become an endemic area for plague. In these natural foci, different species of rodents are present, of which the *Meriones* genus, including *M. libycus*, *M. persicus*, *M. vinogradovi*, *M. tristrami*, plays an important role in the persistence of plague. The latter two species are extremely sensitive to the causative agent of the disease, whereas the first two are resistant. The presence of plague in Kurdistan thus depends on the ecological interplay between susceptible and resistant rodent species living in close contact. Along with these species, other types of rodents are present, such as *Microtus*, *Mesocricetus*, *Allactaga* and *Cristolus*; these rodents are less frequently found and play a minor role in the epizootic evolution of the region [24]. In 1953, a research center was founded in the village of Akanlu, 100 km northwest of Hamadan and on the border of Kurdistan; this center was opened to conduct research on plague and has since been in operation as a surveillance center for plague in western Iran. In that center, Karimi [50] concluded that the plague bacterium can survive in soil for several months.

Marcel Baltazard, the former director of the Pasteur Institute of Iran, and his Iranian colleagues studied 14,102 rodents in this region in 1966 and 1967, and found 66 infected rodents [24]. During this time, nine outbreaks of plague were reported, mostly in Kurdistan, with 156 reported deaths. Evidence showed that outbreaks of bubonic plague in this area, where no domestic rodents are present, were due to inter-human transmission by the flea *Pulex irritans*, starting with rare cases of plague contracted in the fields. Such instances of inter-human transmission of plague, originating in villages, tend to die out rapidly due to the sparse population of the villages, the long distances between them, and the paucity of means of communication. However, when imported into an urban area with a denser human population, plague can immediately become the terrifying disease it was during the Middle Ages [24]. The last formally reported outbreak of human plague in Kurdistan was in 1966 [51].

A major discovery resulting from the studies of these researchers in Kurdistan was the concept of burrowing plague [52]. It was shown that *Y. pestis* could remain alive for several years in the burrows of dead rodents, and then re-infect the new rodents colonizing these empty burrows. In addition to the well-established rodent-flea-rodent cycle, they demonstrated the existence of the burrow-rodent-burrow cycle, allowing the inter-epizootic maintenance of the plague bacillus in its endemic foci [53].

The surveillance of regions surrounding the plague focus in Kurdistan, involving the study of wild rodents, revealed the existence of an epizootic center in Sarab in eastern Azerbaijan Province, north of Kurdistan, where plague had not yet been reported [25]. In this region in 1976, fourteen samples of *Y. pestis* were isolated from wild rodents (*M. persicus*, *M. vinogradovi*, and *Mesocricetus brandti*) and from their fleas (*Xenopsylla conformis* and *Nosopsylla iranus iranus*) [25].

The surveillance of plague in Kurdistan by the Pasteur Institute of Iran revealed that the disease remained silent for a period of between three to five years in its natural foci and then reappeared in its rodent hosts [21,54,55]. The same finding was reported from different parts of the world, including India, Brazil, Argentina, South Africa, and the US [56]. It is clear that the disappearance and reappearance of plague after several years is multifactorial, very complex, and depends on the interactions of its components (rodents, vectors, ectoparasites), population density, life cycles, the geographical distributions of the components, the season, rains, saturation, temperature, and local and global climate fluctuations [57,58].

The monitoring of plague continued from 1978 to 2001 through the Research Center for Emerging and Reemerging Infectious Diseases, located in Akanlu village in Hamadan. It concentrated on monitoring the disease in rodents, and positive rodents and/or fleas were identified. According to studies of the Pasteur Institute of Iran surveillance team during the Iran-Iraq war (1980-1988), in the areas affected by war, no *Y. pestis* infections were observed in the 1,800 rodents and 36,000 fleas monitored [59].

A recent study in 2011 and 2012, conducted at the Research Center for Emerging and Reemerging Infectious Diseases (Akanlu), of the border area between Kurdistan and Hamadan provinces showed that 1.02% of the rodent population and 3.42% of dogs were positive for the antibody against the bacteria, implying that this focus is still active [16].

Because most cases of plague outbreaks occurred before the emergence of microbiology, both the history and the historical population structure of *Y. pestis* are difficult to characterize. Using molecular biology technology on the few available strains from Iran and Kurdistan revealed that all isolates belonged to the Medievalis biovar [60], indicating that they are of Asian origin and are not from the third pandemic. The Orientalis biovar, as the causative agent of the third pandemic, was widespread and has been reported in Turkey [60,61], the northwestern neighbor of Iran. Further investigation using ancient DNA technology [62,63] and studying the population structure of *Y. pestis* in Iran and its neighboring countries will hopefully supply strong evidence to fill the gaps on how the bacterium entered and circulated in Persia (Iran), and which biovar and genotypes of *Y. pestis* affected this region.

## PLAGUE IN THE MIDDLE EAST AND NORTH AFRICA

It is clear that plague frequently recurred in the Middle East and North Africa for over half a millennium, but the dating and the characterization of the nature of the plague outbreaks are fairly imprecise. Nevertheless, an attempt has been made to describe the periodicity and nature of recurrences of plague. The rapidity and the severity of these outbreaks raise the question of the endemicity of plague in this region.

**Figure 2.**
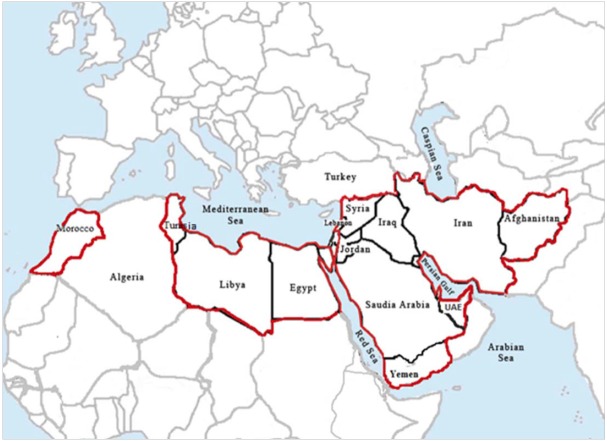
Countries in the World Health Organization Eastern Mediterranean Region affected by plague.

Roughly over the past century, plague outbreaks have been seen in at least 14 countries in the WHO Eastern Mediterranean Region in the Middle East and North Africa (Figure 2); all of these, as well as many earlier outbreaks, are briefly reviewed below.

### Afghanistan

The WHO Expert Committee on Plague determined plague to be endemic in Afghanistan in its report in 1953 [64]. Enzootic sylvatic rodent plague is endemic in the northeast (Badakshan, Konar) along the Pakistani border; isolated cases of human bubonic plague have occurred, particularly along the Pakistani border. Meriones spp. (gerbils) and various Microtinae (meadow mice) are sylvatic reservoirs of plague in this region [65]. In late 2007, an outbreak of plague with acute gastroenteritis symptoms occurred in Nimruz Province in southern Afghanistan; in this outbreak, 17 of the 83 infected cases died, and consumption of infected camel meat was diagnosed as the source of infection [66].

### Bahrain

In 1529 CE, an outbreak of plague occurred in Bahrain; leading to the failure of a Portuguese attack on this region. A plague epidemic in the Arabian littoral also affected Bahrain in 1773 [38,67,68]. From 1907 to 1914, two other epidemics of bubonic plague were reported in this area [67]. In 1914, 1915, and 1924, imported cases of plague were also reported in Bahrain [48].

### Iraq

Throughout its history, Iraq has experienced multiple epidemics of plague [69]. In 716 and 717 CE, a large outbreak known as *al-Ashraf* (*the Notables*) was recorded in Iraq and Syria [70]. In an epidemic of bubonic plague in 1772 and 1773, many victims died in cities such as Basra (with 250,000 deaths) and Mosul. In 1801 CE, a large plague epidemic occurred in Mosul and Baghdad [38,71]. A plague epidemic occurred again in Baghdad in 1908 [33]. From 1923 to 1924, approximately 90 cases of pneumonic plague were reported in Baghdad, and some plague outbreaks were reported in Basra [48,72].

### Jordan

Plague in Jordan was first seen in the seventh century [23]. In 1997, an outbreak of bubonic plague was reported in northeastern Jordan, and all affected cases recovered from the disease. Two dogs tested serologically positive. All cases were infected through the consumption of camel meat [73].

### Kuwait

The plague outbreak of 1773 CE in Iraq spread to Kuwait, leading to an outbreak in this region [68].

### Lebanon

This country has been affected by plague in a variety of ways over the ages and has played a significant role in transmitting the disease to other parts of the world. The plague epidemic in Marseille, France, in 1720 led to 50,000 deaths and occurred via transmission of the infection by a ship from Lebanon [33]. In 1900, four cases of plague were reported in Beirut. This outbreak took place after 65 years with no reported cases [70].

### Saudi Arabia

This country has experienced several outbreaks of plague in the past [33]. An outbreak of plague took place in 1773 CE in the al-Qatif region, an area located in the eastern part of Saudi Arabia [68]. In 1897 and 1898, several fatal cases of plague were reported in Mecca. In 1899, only two cases, both originating in Jeddah, were observed in Mecca [74]. In 1994, five cases of bubonic plague were reported in Goriat, a city located in the desert in southern Saudi Arabia with one reported death. The source was consumption of an infected camel’s liver [75]. The presence of plague in wildlife and domestic animals is confirmed in this country, and this should be considered a potential risk for the general population as well as for Hajj pilgrims [76].

### Syria

The circulation of plague among Syria, Palestine, and Egypt occurred regularly throughout history [77,78]. Accordingly, data related to plague in these three regions have commonly been reported together [70,79]. Outbreaks between 541 and 749 CE in these regions were related to regional trading and the disease was transmitted into Syria via ships entering from Egypt [78]. An outbreak in 746 CE occurred due to a military attack on Constantinople, in which soldiers used swords contaminated with plague [80].

### United Arab Emirates

In 1914 and 1924, imported cases of plague were reported in Dubai [48].

### Yemen

The oldest recorded case of human plague in Yemen occurred near the border with Saudi Arabia, in Asir Province, in 1815, and is believed to have spread to Jeddah and Mecca [81]. Since the 1815 outbreak in this area, few outbreaks have been reported, perhaps due to the lack of investigation. A plague outbreak was reported in Khawlan in the northern highlands of Yemen in 1969 and left 15 dead in two villages [82]. The isolation of *Y. pestis* confirmed the nature of this outbreak in 1969.

### Egypt

In 1347 CE, plague reached Alexandria in Egypt, most likely through the port’s trade with Constantinople and ports on the Black Sea [83]. Between 1347 and 1517, Egypt experienced more than twenty outbreaks. In an outbreak in 1581, approximately 500,000 people died of plague [35]. In the plague pandemic in the sixth century, more than 900,000 cases in Egypt and 22,000 cases in Gaza (Palestine), which was under the authority of Egypt at that time, were reported [80]. In a large outbreak in 1835, approximately 33,000 people died. In 1899, after a period of 50 years with no reported cases, plague recurred in Egypt, and between 1899 and 1919, approximately 15,000 cases of human plague were reported [35,84]. The coexistence of the Nile rat, *Arvicanthis niloticus*, and ectoparasites in combination with trade with Asia, Africa, and the Mediterranean, as well as the Nile floods, suggests that it is possible that *Y. pestis* was primarily a disease of the Nile rat, indicating that Egypt may have been the most probable point of origin of bubonic plague as an epidemic disease [85]. In 1940, 452 cases were reported in Assiut province and some cases were also reported in Port Said. In 1943, an outbreak was observed in the Suez Canal area; and in the Ismailia district and Port Said in 1944 accounting for a total of 862 cases. In 1945, plague caused 19 cases in ports along the canal. In 1947, a 15-case outbreak took place in Alexandria and was the last reported outbreak in Egypt [86]. The reemergence of plague in Egypt should not be excluded, due to the presence of suspected potential natural foci and global climate change.

### Libya

Between 1913 and 1920, multiple outbreaks occurred in Libya, with the largest taking place in 1917 in Benghazi, where 1,449 people died of plague [87]. Twelve confirmed cases were noted in 1939 to 1943 in a locality 12 km from Tripoli [86]. Small outbreaks occurred in 1972, 1976, 1977, and 1984 near Tobruk, close to the border with Egypt. In 2009, after 20 years with no reported cases, the reemergence of plague was observed. Three members of a family were infected with the septicemic form and one of them died. This outbreak was of Asian origin [88,89]. Another possible outbreak of plague comprising more than 20 cases occurred at Tobruk during the Libyan revolution in May 2011 [86]. These data suggest that plague has active foci in Libya.

### Morocco

The first plague cases reported in Morocco were in 1909 among 25 military stations in Casablanca. A severe epidemic with 8,000 to 10,000 deaths took place in 1911. Between 1940 and 1945, almost 5,400 cases were reported in Morocco in the regions of Chaouia, Agadir, Marrakech, Rabat, Doukkala, and Port Lyautey [86,90]. Another large outbreak of plague occurred in 1977, in which almost 50,000 people died [91].

### Tunisia

Plague has not been very prevalent in Tunisia [92], although plague epidemics occurred in Tunisia from 1870 to 1900. Two major plague epidemics also struck Tunisia in 1784 and 1818, which lasted for months [93]. Variable susceptibility to plague has been reported among the flea and rodent species in Tunisia [94]. Twelve cases were reported in 1940 and one in 1941 [86]. A plague outbreak was recorded in 1944, in which 64 people were infected with 27 deaths [95].

## CONCLUSION

According to the WHO Expert Committee on Plague (1959) [96], and field investigations [20,21,53,54], Iranian Kurdistan remains an active plague focus. Recent surveillance in 2011 to 2012 likewise demonstrated that plague is active in this area [16]. Investigations in Turkey, Syria, and Iraq failed to confirm the existence of plague in wild rodents, but an isolated human plague strain in Turkey near the Syrian border [97], with the same biochemical character as the Iranian strains, supports the view that all these foci form part of a large enzootic area [98].

Almost all of the countries in the Middle East and North Africa have reported human plague outbreaks, although less focus has been placed on epidemics of plague in wildlife. In the last 50 years, human plague outbreaks have been reported in Saudi Arabia [75,76], Afghanistan [65,66], Libya [88,89], Morocco [91], Algeria [99,100], and Jordan [73].

The presence of exemplary studies on the wildlife population in Iran may be due to the existence of a research center founded in 1952 as part of the Pasteur Institute of Iran; at this center, extensive studies have been carried out on rodents and other animal hosts of *Y. pestis* in Iran.

Comprehensive studies of locations with historical records of human plague could help clarify the current status of the disease in this region. Otherwise, without adequate knowledge and preparation, human plague outbreaks are expected to continue.

The lack of reports of human plague over the past 50 years does not necessarily imply that the disease has not occurred among humans in Iran. Since Iran has experienced outbreaks of plague for several centuries, neighboring countries have reported the disease in recent years, the disease can be silent for decades, and the circulation of *Y. pestis* has been observed among rodents and dogs in western Iran, more attention should be paid to disease monitoring in areas with previously reported human cases and in high-risk regions with previous epizootic and enzootic activity. Plague should be more strongly emphasized in the medical education system in Iran. It is highly recommended that general practitioners and healthcare workers obtain adequate knowledge of the natural cycle of *Y. pestis* and the clinical signs of plague in order to help them identify the disease. Moreover, further studies are needed to better clarify the epidemiology of plague in Iran.

Finally, the following suggestions may be made to improve the surveillance of plague in Iran: 1) Health staff and practitioners in areas with prior outbreaks of plague, as well as in the eastern and western border areas of Iran, should be trained to be aware of this disease. 2) A standard protocol should be designed for preparing and dispatching suspected samples from all over the country to the Pasteur Institute of Iran. 3) Educational materials on plague in the form of pamphlets, seminars, and conferences concerning zoonotic diseases should be prepared and disseminated. 4) The Research Center for Emerging and Reemerging Diseases of the Pasteur Institute of Iran (Akanlu) should be equipped and empowered as the main center of surveillance, research, and education about plague and other emerging and reemerging diseases in Iran.

## Figures and Tables

**Table 1.** Plague outbreaks in Iran during the 19th and 20th centuries

Outbreak area	Province	Location in Iran	Year	No. of mortalities (no. of case)	Reference
Caspian sea littoral	ND	North	1829-1833	ND	45
ND	Khorasan	Northeast	1829-1833	ND	45
ND	Kurdistan	West	1829-1835	Thousands	27
ND	Kermanshah				27
ND	Hamadan				27
Rasht	Gilan	North	1830-1831	40,000 (in Rasht)	41
ND	Mazandaran		1830-1831	80,000 (in Barforoosh)	40
ND	Azerbaijan	Northwest	1830-1831	30,000 (in Tabriz)	26, 38
Saghez, Baneh Serin Bulagh	Kurdistan	West	1871	Thousands	42, 43
Shushtar	Khuzestan	Southwest	1876	1,800	41
Sabzevar			1877	37	26
			1878	Thousands	27
Rasht	Gilan	North	1877	ND	41,42
Bushehr	Bushehr	South	1877	ND	47
ND	Kermanshah	West	1877	ND	47
ND	Khorasan	Northeast	1877	ND	42
Persian Gulf region	ND	South	1899	ND	45
Around Sistan Lake	Sistan and Baluchestan	East	1906	1,409	44
Bushehr	Bushehr	South	1910	66	44, 45
			1911	98	44, 45
			1912-1913	725 (965)	44, 45
ND	Kurdistan	West	1913	ND	22
Torbat-e Jam	Khorasan	Northeast	1913	ND	22, 45
Bandar Lengeh	Hormozgan	South	1914	0 (1)	48
Bandar Abbas			1915	0 (3)	48
Khorramshahr	Khuzestan	Southwest	1915	0 (3)	45
Khorramshahr			1917	43 (79)	45
Abadan			1917	409 (481)	45
Torbat-e Jam	Khorasan	Northeast	1921	ND	24, 45
Kariz					
Khorramshahr	Khuzestan	Southwest	1922	ND	48
Chabahar	Sistan and Baluchestan	East	1923	ND	48
Khorramshahr	Khuzestan	Southwest	1923	45 (71)	48
Abadan	Khuzestan	Southwest	1923	409 (481)	48
Bandar Lengeh	Hormozgan	South	1923	0 (4)	48
Khorramshahr	Khuzestan	Southwest	1924	115 (152)	44, 48
Abadan	Khuzestan	Southwest	1924	0 (233)	44, 48
Genaveh	Bushehr	South	1924	14 (17)	48
Bandar Abbas	Hormozgan	South	1924	7 (12)	48
Bushehr	Bushehr	South	1924	0 (1)	48
Bandar Lengeh	Hormozgan	South	1924	0 (1)	48
Aghbolagh Morshed	Kurdistan	West	1947	56	45
Sameleh and Sarbaghleh	Kurdistan	West	1947	17	45
MazidAbad	Kurdistan	West	1951	2	45
Gozar-darreh	Kurdistan	West	1952	45	45
Gavmichan	Kermanshah	West	1952	8	45
ZenalKandi	Western Azerbaijan	Northwest	1958	6	45
Ghaderabad	Kurdistan	West	1961	7	45
Sarumal	Kurdistan	West	1963	14	45
SeyyedAbad	Kurdistan	West	1966	1	45

ND, not determined.
